# Candidalysin at the epithelial-systemic interface: mechanistic evidence, critical-care relevance, and translational opportunities in invasive candidiasis

**DOI:** 10.3389/fcimb.2026.1918325

**Published:** 2026-08-12

**Authors:** Yidan Fu, Xiaonan Li, Xianyao Wan

**Affiliations:** Department of Critical Care Medicine, The First Affiliated Hospital of Dalian Medical University, Dalian, China

**Keywords:** biomarker, candida albicans, candidalysin, candidemia, critical care, epithelial barrier, invasive candidiasis, virulence-directed therapy

## Abstract

Invasive candidiasis remains a stubborn source of morbidity and mortality in critically ill and immunocompromised patients. One reason progress has been slow is that the route from epithelial colonization to bloodstream or deep-organ infection is still described with more confidence than the evidence often allows. Candidalysin, a 31-amino-acid amphipathic peptide released from the Ece1 precursor by *Candida albicans* hyphae, is well established as a driver of epithelial damage and mucosal immune activation. Its contribution to invasive disease is far less certain. Recent work has broadened the molecular framework for candidalysin biology. Host-binding studies have identified sulfated glycosaminoglycans and other candidate partners. Biophysical studies have refined models of peptide polymerization, membrane insertion, and host membrane repair. Infection models have linked candidalysin to intestinal epithelial barrier failure, catheter persistence, macrophage escape, neutrophil remodeling, commensal fitness, and protective Th17 immunity. These observations sit at different distances from human invasive candidiasis: some are biochemical or cell-based, some are supported by organotypic or animal models, and only a small subset currently has human diagnostic or clinical correlation. In this review, we separate these evidence layers and argue that candidalysin should be treated as a candidate biomarker or adjunctive target for selected toxin-producing *Candida albicans* infections, not as a general explanation for invasive candidiasis.

## 1 Introduction: why candidalysin needs careful clinical reassessment

In this review, invasive candidiasis (IC) refers to candidemia and deep-seated Candida infection of normally sterile tissues. Candidemia denotes bloodstream infection, whereas deep-seated IC refers to sterile-site infection with or without positive blood cultures. We use mucosal or cutaneous candidiasis for infection confined to epithelial surfaces. IC is familiar to intensivists, transplant physicians, hematologists, and surgeons. Clinically, IC appears when more than one barrier fails. The relevant barriers include epithelial surfaces injured mechanically or chemically, endothelial and device-associated interfaces breached by intravascular catheters, and immune barriers weakened by neutropenia, corticosteroids, or critical-illness immune dysfunction. Severe IC remains a major cause of fungal mortality worldwide (Brown et al., 2012; Denning, 2024). The diagnostic problem is equally persistent. Blood culture misses a proportion of deep-seated disease. Delayed active treatment worsens outcomes (Cornely et al., 2025; Lass-Flörl et al., 2024; Pappas et al., 2016). The 2025 global guideline therefore keeps the emphasis on rapid diagnosis and source control. It also emphasizes species identification, susceptibility testing, and early active systemic antifungal therapy (Cornely et al., 2025).

The discovery of candidalysin in 2016 changed the language of Candida pathogenesis. For the first time, *C. albicans* had a defined peptide toxin that could directly damage host membranes (Moyes et al., 2016). Subsequent work connected candidalysin to oral and vaginal candidiasis and to epithelial danger sensing (Naglik et al., 2019; Richardson et al., 2018; Russell et al., 2023). Other studies linked the toxin to macrophage escape and neutrophil activation (Kasper et al., 2018; Lin and Filler, 2025; Lortal et al., 2025). That success has created a second problem. Evidence from synthetic-peptide exposure, epithelial monolayers, and mucosal infection models is sometimes extrapolated beyond its experimental context to imply systemic causality in human candidemia. That causal link has not been established. IC is heterogeneous. Non-albicans species matter, and dissemination can begin in the gut, a catheter, an abdominal focus, or an iatrogenic breach. A more precise question is therefore: in which forms of *C. albicans* IC is candidalysin likely to be causal, measurable, and therapeutically actionable?

Several recent reviews already summarize pore formation, host targets, and cell activation in detail (Lin and Filler, 2025; Lortal et al., 2025; Russell et al., 2023). We do not duplicate them here. This review instead asks how far candidalysin biology can be translated into invasive-disease diagnosis or therapy, and where the evidence still remains indirect.

This article is a narrative review rather than a systematic review. We prioritized primary mechanistic studies, recent reviews, clinical guidelines, and emerging diagnostic or therapeutic studies identified through focused PubMed searches and reference tracking. Throughout the review, we distinguish biochemical and cell-based evidence, organotypic and animal-model evidence, and human observational evidence, because these evidence types support different levels of clinical inference. When evidence types point in different directions, we give human disease-linked data priority for clinical claims and mechanistic data priority for biological plausibility. A pathway that is strong in peptide, cell, or animal systems but weakly connected to patients is therefore framed as a plausible mechanism, not a validated clinical target. Conversely, a clinical association without molecular resolution is treated as a risk signal that still needs mechanistic testing.

## 2 Biogenesis, sequence, and pore-forming behavior

### 2.1 From ECE1 transcription to mature candidalysin activity

Current consensus identifies candidalysin as a 31-amino-acid amphipathic alpha-helical peptide released from the Ece1 precursor (Lortal et al., 2025; Moyes et al., 2016; Müller et al., 2024).

*ECE1* expression is usually associated with filamentation, but it should not be used as a shorthand for filamentation itself. Hyphae bring mechanical penetration, adhesins, secreted enzymes, and a delivery geometry; candidalysin adds membrane permeabilization and signal amplification. Experiments that collapse these features into a single “hyphal virulence” category risk over-crediting or under-crediting the toxin. Mutant-complementation studies should separate loss of *ECE1*, loss of the candidalysin-encoding segment, defective processing, and broader defects in hyphal fitness.

### 2.2 Polymerization, membrane insertion, and host repair

Biophysical studies indicate that candidalysin self-assembles in aqueous solution into polymers and loop-like structures before membrane insertion. Recent work refined this polymerization mechanism and linked oligomerization to pore formation (Russell et al., 2022, 2023; Schaefer et al., 2024). Membrane permeabilization produces potassium efflux and calcium influx. It also disrupts ionic homeostasis and remodels the cytoskeleton. Above a concentration-time threshold, these events progress to lytic damage. This is not simply a static protein pore. Peptide concentration, lipid composition, pH, ionic environment, and sequence variance all influence activity.

The host side of the interaction is equally active. Calcium-dependent repair, MEK-dependent responses, annexins A1/A2/A6, and extracellular-vesicle shedding help remove candidalysin-damaged membrane. Patch repair and ceramide pathways appear less protective than they are against several bacterial pore-forming toxins (Thapa et al., 2026). These observations describe a contest between injury and repair. Low or transient exposure can produce signaling without immediate lysis, whereas sustained delivery in an invasion pocket can overwhelm repair and become tissue damage (Mogavero et al., 2021).

### 2.3 Receptors and sequence variation expand the biological range

A global fungal-host interactome identified many candidate binding partners, and later work placed sulfated glycosaminoglycans among the epithelial targets that promote candidalysin binding and toxicity (Lin et al., 2024; Zhang et al., 2024). The older lipid-disruption model proposed that candidalysin acts mainly as an amphipathic peptide that binds lipid bilayers, self-associates, inserts into host membranes, and creates membrane lesions that permit calcium influx and epithelial damage. That model still holds, but it is no longer sufficient on its own. The glycan data add an upstream host-surface step: sulfated glycosaminoglycans can increase epithelial binding and toxicity, consistent with local concentration of the peptide before lipid engagement. Whether these glycans also orient candidalysin for membrane insertion remains less directly resolved. Even so, they change the efficiency of membrane engagement and lesion formation rather than replacing the lipid-interaction step. Natural sequence variation adds another layer, because candidalysin variants differ in membrane interaction, calcium influx, cytotoxicity, MAPK activation, and cytokine output (Wickramasinghe et al., 2024). For clinical studies, *ECE1* positivity is necessary, but not sufficient, to infer biologically relevant candidalysin activity. It supports the genetic capacity to produce the toxin, but sequence, expression, secretion, and functional activity all matter.

## 3 The epithelial-to-systemic continuum

### 3.1 Epithelial injury and danger signaling

**Figure 1:**
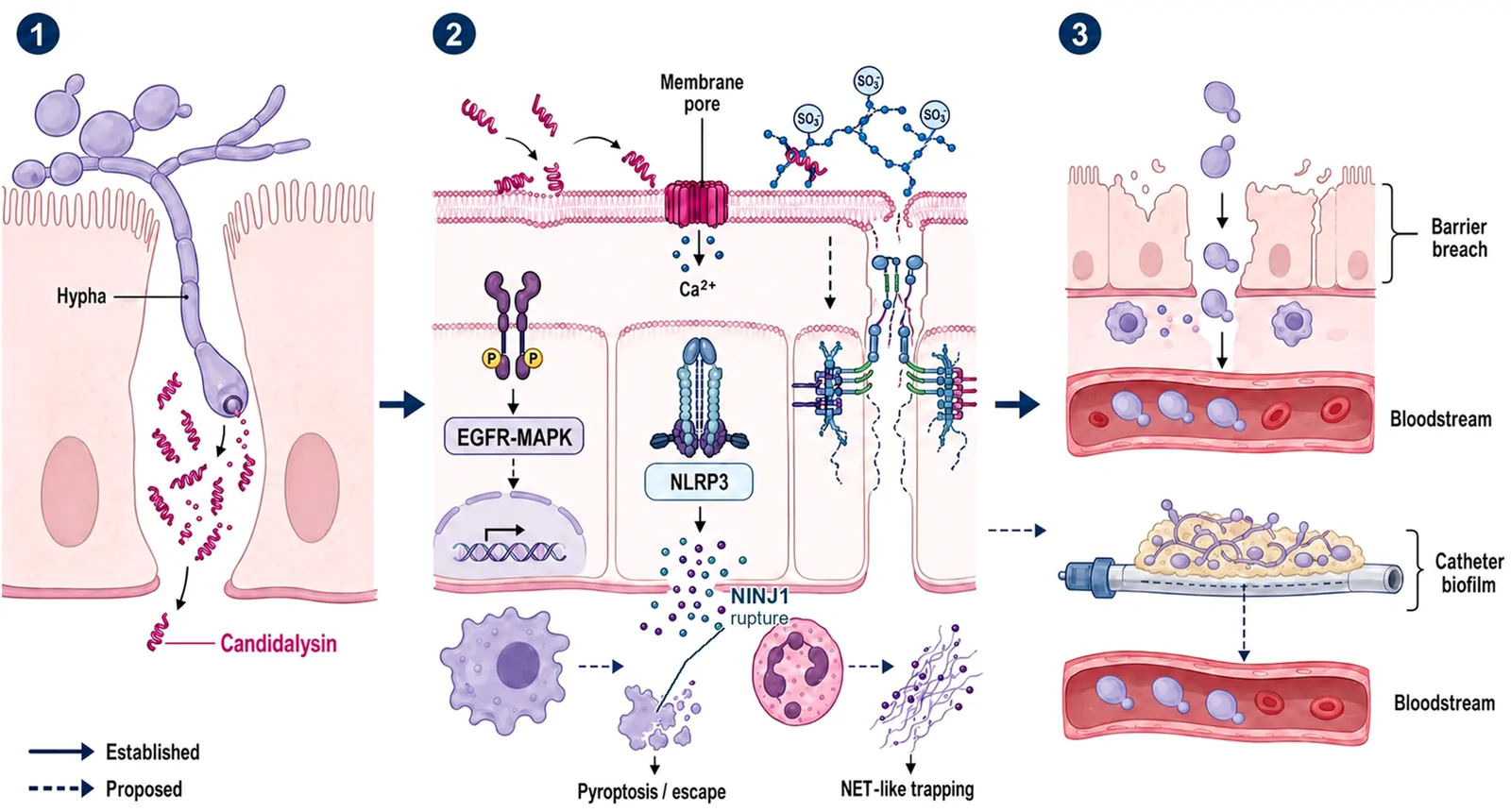
Candidalysin across the epithelial-systemic interface. Hyphal secretion concentrates candidalysin at invasion pockets. At these sites, the peptide can engage host membranes, form pores, induce calcium influx, activate EGFR-MAPK and inflammasome signaling, and contribute to junction failure. Step 2 also includes NINJ1-dependent macrophage rupture as a proposed branch. Solid arrows denote mechanisms supported directly in the indicated experimental context; dashed arrows emphasize extrapolation or context dependence. Barrier damage can permit yeast translocation, whereas catheter biofilms provide a separate intravascular route. The image does not imply that circulating candidalysin has been established in human candidemia. Symbols and abbreviations in the image are defined here for standalone reading: SO3-, sulfate groups on sulfated glycosaminoglycans; Ca2+, calcium ions; EGFR, epidermal growth factor receptor; MAPK, mitogen-activated protein kinase; NLRP3, NLR family pyrin domain containing 3; NINJ1, ninjurin-1.

At mucosal and other epithelial surfaces, candidalysin activates a damage-response network centered on EGFR, MAPK signaling, c-Fos, and MKP1 (Ho et al., 2019; Moyes et al., 2016). This network has been described in oral, vaginal, and intestinal contexts. NF-kappaB engagement and cytokine release vary by epithelial site and experimental system. EGFR activation is not a simple toxin receptor event. Candida adhesin-mediated invasion, candidalysin-induced damage, receptor trafficking, and host ligand release intersect. A 2026 study showed *ECE1*- and *ALS3*-dependent EGFR ubiquitination and altered receptor degradation. These data support the view that fungal invasion actively remodels host signaling rather than simply triggering it (Lortal et al., 2026). This local hyphal invasion and toxin-delivery phase is shown schematically (**Figure 1**, step 1).

Tight-junction disruption and barrier permeability are the products of several insults arriving together: hyphal penetration, candidalysin exposure, host-cell death, and inflammation. Gut-on-chip and organoid-tubule systems now reproduce several candidalysin-dependent epithelial phenotypes (Morelli and Queiroz, 2025). These include loss of transepithelial resistance, actin remodeling, lactate dehydrogenase release, cytokine output, and LL-37 induction. These systems are a real advance over peptide-treated monolayers. They remain simplified representations of critical illness. They do not fully capture vascular leakage, immune paralysis or hyperinflammation, antimicrobial exposure, nutritional changes, microbiome injury, or concurrent antifungal therapy. The epithelial membrane-injury and signaling layer is summarized in the central panel (**Figure 1**, step 2).

### 3.2 Intestinal colonization, translocation, and the commensalism paradox

The gastrointestinal tract is a major reservoir for *C. albicans* and a plausible origin of IC when antibiotics, mucosal injury, dysbiosis, parenteral nutrition, surgery, or immune suppression converge. Candidalysin can assist intestinal epithelial barrier failure, but bloodstream entry is not equivalent to hyphal penetration alone. Allert et al. showed that fungal translocation across intestinal epithelia requires invasion, epithelial damage, and loss of barrier integrity, with candidalysin acting as a key damage factor (Allert et al., 2018). More recently, Sprague et al. directly tested the yeast-cell question and reported that yeast cells can passively translocate with invasive hyphae when host-cell damage is present, including damage facilitated by candidalysin (Sprague et al., 2025). This provides a plausible working model for how tissue-invasive hyphae could coexist with the yeast-dominant morphology commonly recovered from blood, but direct confirmation in human IC remains limited. The barrier-breach and bloodstream-entry outcome is shown in the right panel (**Figure 1**, step 3).

Candidalysin is not only a damage factor. In microbiota-replete mice, hypha-associated factors including candidalysin improve early gut colonization fitness, partly through antibacterial effects; later, adaptive IgA selects against hyphal morphotypes (Liang et al., 2024). Earlier oral-infection work showed that candidalysin helps epithelial cells orchestrate innate type 17 responses through IL-1- and IL-17-linked signaling (Verma et al., 2017). More recently, intestinal exposure to candidalysin promoted yeast-specific Th17 differentiation and protection against subsequent disseminated infection (Nguyen et al., 2026). The same peptide can help colonization, injure a compromised epithelial barrier, and prime protective immunity. Which of these dominates depends on dose, location, microbial ecology, and host state.

Recent colonization studies sharpen this point. Dynamic *ECE1* expression allows *C. albicans* to use transient hyphal growth during oral colonization without maintaining uniformly high toxin output, whereas gastrointestinal colonization in mice depends on a coordinated program that includes candidalysin and adhesins (Fróis-Martins et al., 2025; Mauk et al., 2025). A binary “toxin on/toxin off” model does not fit these observations. If a candidalysin-based biomarker becomes clinically useful, the assay will need to reflect active toxin secretion at a particular anatomical site. Detecting only the genetic capacity to produce the toxin is unlikely to be sufficient.

### 3.3 Multi-site epithelial colonization: risk signal or proven source?

Candida colonization is not confined to the gastrointestinal, oral, or reproductive mucosa. Candida species can also colonize the skin. The cited atopic dermatitis studies support increased *Candida albicans* colonization or Candida-specific humoral responses in affected patients, whereas the psoriasis literature is stronger at the Candida genus level and should not be overread as *C. albicans*-specific or candidalysin-specific evidence (Javad et al., 2015; Pietrzak et al., 2018; Storz et al., 2024). This expands the anatomical frame beyond mucosal sites alone, but it does not establish a skin-to-bloodstream candidalysin pathway. The clinically relevant question is whether colonization across several epithelial surfaces marks a host state that increases invasive risk, or whether any specific epithelial site directly seeds a particular IC episode.

For classic *C. albicans* IC, the answer remains incomplete. In ICU cohorts, multifocal Candida colonization and Candida score-type approaches are useful risk markers for invasive candidiasis (León et al., 2006, 2009; Pittet et al., 1994). Their predictive value is strongest when colonization occurs together with surgery, parenteral nutrition, severe sepsis, broad-spectrum antibiotics, or central venous access. These tools do not usually identify which epithelial site seeded the bloodstream. Gut injury remains the most plausible route for endogenous candidemia in many critically ill patients, supported by colonization-risk studies and by experimental intestinal-barrier models (Allert et al., 2018; Lass-Flörl et al., 2024; Pittet et al., 1994). Oral, genital, or cutaneous colonization may instead mark a host state permissive for fungal overgrowth.

*Candida auris*, now also referred to as *Candidozyma auris*, illustrates how colonization and healthcare exposure can converge with invasive infection risk (Garcia-Bustos et al., 2026). However, no study has yet shown that candidalysin drives the transition from cutaneous colonization to invasive candidiasis. This remains an area for future research, and patients with inflammatory skin disease should be considered as a prespecified subgroup in studies integrating multisite colonization, strain typing, *ECE1* expression, toxin detection, and clinical source attribution.

### 3.4 Macrophage death and fungal escape

After phagocytosis, *C. albicans* filamentation and candidalysin contribute to macrophage damage and escape. Candidalysin acts upstream of the canonical inflammasome pathway by activating NLR family pyrin domain containing 3 (NLRP3) and promoting caspase-1-dependent processing of IL-1beta (Broz and Dixit, 2016; Kasper et al., 2018). In this host pathway, inflammatory caspases process gasdermin D (GSDMD). GSDMD pore formation then contributes to pyroptosis and fungal escape (Ding et al., 2021). Parallel cell-death routes also operate, and macrophage rupture is not fully explained by pyroptosis (Olivier et al., 2022).

NINJ1, or ninjurin-1, is a host membrane protein that mediates terminal plasma-membrane rupture during lytic cell death (Kayagaki et al., 2021). This matters for Candida infection because it separates upstream death signaling from the final physical rupture of the macrophage membrane. A 2026 study connected this pathway to fungal escape through metabolic membrane rupture. Candida-induced glucose starvation activated NINJ1, which mediated macrophage lysis independently of canonical death programs. Alanine limited NINJ1 oligomerization, and NINJ1-dependent rupture acted together with candidalysin to permit fungal egress (Weerasinghe et al., 2026). These observations argue against single-pathway models of phagocyte death. Blocking NLRP3 alone may not preserve phagocyte integrity, and preserving phagocyte integrity may not automatically improve fungal clearance. The macrophage escape route, including NINJ1-dependent rupture, is shown as a proposed branch within the mechanistic layer (**Figure 1**, step 2).

Macrophage behavior is also shaped by inhibitory checkpoint signaling. In an oropharyngeal model, candidalysin induced macrophage Tim-3 and suppressed antifungal activity (Liu et al., 2025). The result is preliminary and not yet ready for translation. It comes from a mucosal infection model, and checkpoint modulation in IC could either restore antifungal function or aggravate cytokine injury. The answer is likely to differ between liver, spleen, kidney, lung, and intestinal compartments.

### 3.5 Neutrophils, NET-like structures, and intravascular devices

The neutrophil response is similarly two-sided. The toxin can induce NADPH oxidase activity, calcium influx, PAD4-dependent hypercitrullination, and compact NET-like structures that restrict fungal growth partly through zinc deprivation (Unger et al., 2023). Yet in intravascular-catheter models, candidalysin-regulated NETosis supported *C. albicans* persistence, linking a toxin-dependent phenotype to a clinically important route of candidemia (Tseng et al., 2024). Biofilm extracellular vesicles can also carry active candidalysin and stimulate epithelial cytokine responses without necessarily causing overt cytolysis (Lee et al., 2025). Location and delivery vehicle may therefore be as important as total peptide production.

## 4 Current evidence in invasive candidiasis

The supporting evidence is uneven across levels of biological and clinical inference. Biochemical and mucosal data are the most consistent. Intestinal translocation, catheter, phagocyte, and systemic mouse models occupy an intermediate level. Human data remain limited. Candidalysin-deficient strains alter virulence and neutrophil recruitment in murine systemic infection (Swidergall et al., 2019), but intravenous inoculation bypasses the barrier-breach step and cannot tell us whether toxin neutralization would help naturally acquired IC. Gut and catheter models move closer to the clinic, but they still capture only fragments of ICU heterogeneity.

At present, the most developed clinical signal is diagnostic, not therapeutic. Luo et al. tested serum anti-candidalysin IgG in a preliminary pediatric cohort. The stricter comparison used proven IC cases only (n = 20) against non-IC controls (n = 105). It yielded an area under the receiver-operating-characteristic curve of 0.818 (95% CI, 0.736-0.899), with 80.0% sensitivity and 73.3% specificity. The broader comparison combined proven and probable IC cases (n = 77) against the same controls. This choice increased event numbers and better resembled the population in which a screening assay might first be tested, but it also mixed diagnostic certainty levels. In that comparison, the area under the curve was 0.870 (95% CI, 0.821-0.919), with 87.0% sensitivity and 70.5% specificity (Luo et al., 2025).

Both estimates are useful, but for different reasons. The proven-only result is the cleaner diagnostic estimate, whereas the combined result is more clinically pragmatic but less strict in case definition. These numbers are encouraging, but they are not yet practice-changing. The pediatric study included non-IC controls and healthy individuals, but background anti-candidalysin IgG levels in healthy adults, colonized ICU patients, and patients with repeated mucosal Candida exposure remain poorly defined. Antibody kinetics may vary by age, immunosuppression, colonization burden, and previous mucosal Candida infection. Infecting species and cross-reactivity could also move the estimate. Adult ICU cohorts with predefined sampling, age-aware interpretation, and external validation are the minimum next step.

### 4.1 Criteria for target validation in invasive disease

Before efficacy trials, a candidalysin-directed intervention should clear four target-validation hurdles. This framing is adapted from general drug-development and antivirulence-therapy principles (Cegelski et al., 2008; Cook et al., 2014). Those principles require disease-relevant target biology, measurable target engagement, pharmacodynamic effect, safety, and patient-context selection before clinical testing. The infecting isolate must produce biologically active toxin under conditions resembling the patient’s infection. Toxin activity must be detectable in the relevant compartment, or reliably inferred from a validated response biomarker. Neutralization should reduce tissue injury without increasing fungal burden in models that include active standard-of-care antifungal therapy. Finally, the effect should survive clinically realistic variation in strain background, immune state, and route of infection.

This distinction between causal mediator and correlated virulence marker is not academic. *ECE1* expression rises during hyphal growth, and hyphae also express adhesins, invasins, hydrolytic enzymes, and metabolic programs. An association between *ECE1* and severe disease may identify an invasive fungal state without showing that candidalysin is the dominant effector. Cleaner tests already exist in the candidalysin literature. Moyes et al. used *ECE1* deletion, *ECE1* complementation, deletion of the Ece1-III/candidalysin region, synthetic Ece1-III peptide exposure, and peptide variants to separate toxin activity from the wider hyphal program. Richardson et al. extended the mutant-and-peptide logic in vaginal epithelial and mouse infection models. Mogavero et al. then used a mutant panel, forced *ECE1* expression, peptide detection, and nanobody localization to show that delivery into the invasion pocket is required for full epithelial damage (Moyes et al., 2016; Richardson et al., 2018; Mogavero et al., 2021). Clinical studies should be equally cautious about reading anti-candidalysin antibodies as evidence of ongoing toxin-mediated injury.

The right model depends on the proposed clinical niche. Intravenous challenge is useful for organ-phase virulence but bypasses colonization, barrier breach, and device entry. Gut-translocation models more closely approximate endogenous candidemia than intravenous challenge models. Their interpretation, however, depends heavily on experimental conditions. Key variables include the antibiotic regimen, diet, microbiota composition, degree of epithelial injury, and host immune status. Catheter models capture biofilm persistence and local intravascular immunity but may not generalize to intra-abdominal candidiasis. If an adjunct works in two route-specific models, especially with antifungal therapy in the background, the translational claim becomes much stronger.

## 5 Critical-care implications

### 5.1 Candidalysin is not a substitute for current IC management

Current management of suspected or confirmed IC remains anchored in early active antifungal therapy, source control, and microbiological confirmation, including current critical-care guidance for preventive and empirical therapy where appropriate (Epelbaum et al., 2025).

Fluconazole remains useful, but mainly for selected stable patients who are unlikely to have fluconazole-resistant Candida, or as step-down therapy after clinical stability, isolate susceptibility, and bloodstream clearance are established (Pappas et al., 2016). Deep foci, catheter-associated infection, intra-abdominal disease, endocarditis, endophthalmitis, and central nervous system disease require syndrome-specific source control and drug-penetration decisions. Rezafungin has shown non-inferiority to caspofungin in candidemia/IC and offers once-weekly dosing (Thompson et al., 2023). Pooled ICU analyses support comparable efficacy and safety, but this is better antifungal pharmacology, not toxin-directed treatment (Honoré et al., 2024).

A second practical limitation is species. Candidalysin was first characterized in *C. albicans*, but similar structural and functional orthologs have also been identified in *C. dubliniensis* and *C. tropicalis* (Richardson et al., 2022). Even in these toxin-bearing lineages, activity requires the right morphologic and transcriptional state. Candidalysin cannot explain IC caused by *C. parapsilosis*, *Candida auris* (*Candidozyma auris*), *Candida glabrata* (*Nakaseomyces glabratus*), or other important species. *C. parapsilosis* is especially relevant to catheter-associated and healthcare-associated disease, yet its virulence is not organized around candidalysin-like toxin biology (Kullberg and Arendrup, 2015; Lass-Flörl et al., 2024; Trofa et al., 2008). That matters because ICU epidemiology varies geographically and because *C. albicans*, *C. glabrata* (*N. glabratus*), and other non-albicans Candida species are recognized global-priority pathogens (Katsipoulaki et al., 2024). A candidalysin-directed adjunct would therefore need rapid species or toxin confirmation and would likely be most relevant when barrier damage or *C. albicans* device biology is central.

### 5.2 A plausible ICU use case

The most defensible ICU niche is a narrow one: an enriched, biomarker-guided adjunct for high-risk patients with *C. albicans* IC, evidence of ongoing toxin production, and persistent tissue injury or immune dysregulation despite active antifungal therapy and source control. Gastrointestinal epithelial barrier failure with deep-seated candidiasis, selected catheter-associated infections, and severe epithelial-to-systemic *C. albicans* infection are plausible starting points. Even there, neutralization must avoid blunting protective neutrophil or Th17 responses.

### 5.3 Diagnostic development: from association to clinical utility

Diagnostic development should begin from the intended clinical use, and choose an assay platform only afterward. A rule-out test for high-risk ICU patients needs very high sensitivity and fast turnaround. A companion diagnostic for neutralizing therapy needs specificity for active, clinically relevant toxin production. A prognostic test must add information beyond species, source, organ dysfunction, and treatment timing. One anti-candidalysin IgG threshold is unlikely to do all three jobs. Serial sampling of anti-candidalysin IgG in patients with suspected or confirmed IC is feasible in principle, but it needs prospective testing. Such studies should first define baseline distributions in healthy adults and colonized high-risk patients, because high background exposure could reduce the value of a single cutoff. A change over time may be more informative than a single value if antibody dynamics capture recent exposure, while direct peptide or functional assays capture current toxin activity.

A feasible first trial framework should start with the minimum elements needed to test clinical relevance. Consecutive high-risk patients should be enrolled before final disease classification, with prespecified groups that include candidemia, deep-seated IC with negative blood cultures, heavy colonization without invasion, non-albicans candidiasis, bacterial sepsis, and healthy controls. The core sampling plan could be simple: serial plasma collection, standard clinical cultures, source documentation, species identification, and paired testing for anti-candidalysin IgG, direct toxin signal, or a validated functional readout. The key analysis would ask whether candidalysin-related measurements add information beyond species, infection source, antifungal timing, and illness severity. When clinically obtained catheter tips, mucosal swabs, or tissue samples are available, they could be used in nested substudies to test compartment tracking. Validation should then proceed in staged cohorts, beginning with adult ICU patients and expanding to pediatric, neutropenic, surgical, and geographically distinct groups.

The analytical challenges are considerable. Candidalysin is a small amphipathic peptide, and that creates several measurement risks. Adsorption to surfaces, proteolysis, matrix interference, transient exposure, and binding to antibodies or glycosaminoglycans may all distort the signal. Mass spectrometry has been valuable for mechanistic work on candidalysin processing, but that is not the same as a clinical diagnostic assay. Targeted detection of a 31-residue toxin at clinically relevant concentrations in plasma, catheter material, or tissue fluid is likely to be harder than confirming precursor processing *in vitro*. Targeted mass spectrometry, immunoassays, aptamers, and functional membrane-permeabilization readouts should therefore be cross-validated rather than treated as interchangeable. A high-affinity candidalysin aptamer detected by a dual colorimetric-SERS platform is a useful analytical feasibility signal, but diagnostic accuracy in clinical IC remains unestablished (Müller et al., 2024; Sun et al., 2026).

## 6 Translational strategies

### 6.1 Neutralize the mature peptide

Direct neutralization has a clear appeal: it targets toxin-mediated damage rather than fungal viability, so its selection profile may differ from that of fungicidal or fungistatic drugs. It should not be treated as resistance-neutral. Candidalysin is still a pathogen-derived target, and sustained blockade could in principle select altered toxin sequence, expression, processing, secretion, or compensatory virulence programs. Development should therefore monitor fungal burden, antifungal susceptibility, *ECE1* variation, and toxin activity alongside tissue-protection endpoints (Lee et al., 2021; Liu et al., 2021). Anti-candidalysin nanobodies localize to invasion pockets and reduce epithelial damage, cytokine signaling, and neutrophil recruitment in preclinical vulvovaginal models (Valentine et al., 2024). Passive mucosal immunization with monoclonal antibodies targeting candidalysin and Hyr proteins also attenuated experimental vaginal candidiasis. In that study, the inclusion of Hyr targets should be interpreted as a combined anti-virulence immunization strategy rather than evidence that candidalysin neutralization alone is sufficient (Auffray et al., 2025). Heparin can bind candidalysin and neutralize oral epithelial cytotoxicity, but systemic anticoagulant effects make unfractionated heparin a poor template for broad dosing (Ikegami et al., 2023). Safer scaffolds could include non-anticoagulant glycosaminoglycan mimetics, stable peptides, aptamers, or engineered antibodies.

The difficult part is delivery. Neutralizers would need to reach a rapidly secreted peptide at high local concentration inside invasion pockets, survive proteolysis, penetrate tissue, and avoid dismantling useful host defense. Local delivery may be enough for mucosal candidiasis; deep-seated infection may require systemic exposure. Either way, pharmacodynamic assays that distinguish free from bound candidalysin will be essential.

A candidalysin-neutralizing peptide has also been placed into a macrophage-targeted nanomodulator in a colorectal cancer model, where it reduced toxin-associated pyroptosis and enhanced immunotherapy (Yan et al., 2025). The platform is technically sophisticated, but that does not make it antifungal-ready. The indication, delivery system, and tumor immune context are far from IC. For invasive fungal disease, a simpler agent with predictable exposure, manufacturability, and compatibility with echinocandins or azoles may be the more realistic first step.

### 6.2 Suppress production or delivery

Upstream strategies include inhibiting hyphal regulatory circuits, *ECE1* transcription, precursor processing, secretion, or extracellular-vesicle delivery. A Streptomyces-derived methoxy-apo-enterobactin was reported to suppress candidalysin production and *C. albicans* virulence phenotypes (Kim et al., 2025). Such compounds may reduce several hypha-associated traits simultaneously, but that breadth complicates target attribution and safety assessment. Compared with neutralizing a released toxin, blocking morphogenesis or toxin production interferes with a broader pathogen program. Resistance or pathway bypass should therefore be assumed possible rather than exceptional (Lee et al., 2021).

### 6.3 Protect host membranes and tune, not silence, immunity

Host-directed strategies are tempting because the same injury pathways recur across models. Relevant pathways include membrane repair, glycosaminoglycan binding, EGFR trafficking, inflammasome signaling, NINJ1 oligomerization, metabolic stress, and NETosis. None is a clean on-off target. EGFR signaling can support epithelial responses, but it may also facilitate invasion. NLRP3 and GSDMD contribute to lysis, yet they also generate antifungal cytokines. Repurposed second-generation sulfonylureas can dampen this pathway *in vitro* (Lowes et al., 2020). NETs restrict fungi, but they can also promote immunothrombosis and catheter persistence. Alanine rescue of macrophage integrity is mechanistically interesting, but it still needs validation in sepsis physiology. If host-directed therapy is pursued, it should be time-limited and compartment-aware. It should also be paired with effective fungal killing, while recognizing that background antifungal therapy is itself a resistance-selecting pressure that must be monitored in combination programs (Lee et al., 2021). This caution is necessary because intact innate and Th17 responses remain essential for controlling Candida (Netea et al., 2015).

### 6.4 Biomarkers and vaccination

A candidalysin biomarker would be clinically useful only if it informs a defined decision: whether a Candida-positive patient is colonized or has invasive disease, whether antifungal therapy should be initiated or escalated in a high-risk patient, whether a patient is eligible for toxin-directed adjunctive treatment, or whether de-escalation is reasonable when IC becomes unlikely. It could also provide a pharmacodynamic readout. Candidate analytes include circulating peptide, *ECE1* transcripts, functional pore-forming activity, and anti-candidalysin antibodies. Antibody measurements may underperform in neutropenia, hypogammaglobulinemia, or very early infection. The vaccine question is even more delicate. Candidalysin can prime protective Th17 immunity (Nguyen et al., 2026), so a vaccine strategy is biologically plausible. One possible route would be a toxoid-like approach, analogous in concept to detoxified bacterial toxin vaccines such as tetanus toxoid. For candidalysin, this would require engineering or selecting a peptide form that loses cytolytic activity while retaining useful antigenicity, followed by careful adjuvant design to avoid tissue injury or immunopathology.

### 6.5 Development risks and combination principles

Virulence-directed therapy should also not be assumed to be resistant to evolutionary escape. Candidalysin blockade could still select altered *ECE1* sequence, higher expression, compensatory adhesins or hydrolases, different morphologic programs, or a shift toward device-associated persistence. Resistance monitoring should include whole-genome sequence, *ECE1* expression, and peptide activity, not minimum inhibitory concentration alone. Ecology matters as well. In GI colonization models, candidalysin promotes fungal commensal fitness partly through antibacterial effects against competing bacteria (Liang et al., 2024). Neutralizing or inhibiting the peptide could therefore relieve this antimicrobial pressure and reshape the commensal microbiome, with uncertain effects on colonization resistance or recurrence risk.

Combination studies should be built around the biology of complementarity. Antifungals reduce viable burden; toxin neutralization limits new membrane injury; source control removes a protected niche. Resistance and escape should be treated as shared development endpoints: antifungals can select drug resistance, whereas toxin neutralization could favor altered or toxin-independent virulence. Showing additivity in a peptide-only assay will not be enough. Experiments should compare simultaneous and staggered dosing, measure viable fungal burden and tissue damage separately, and include drug-resistant isolates. A useful adjunct should improve host-centered outcomes without weakening fungicidal activity, delaying clearance, or creating clinically important immunosuppression.

## 7 A translational roadmap

Four advances would make the field more clinically convincing. The current evidence map and development priorities are summarized in **Tables 1**, **2**. First, clinical-strain studies should connect *ECE1* sequence, expression, secreted peptide abundance, and functional activity instead of treating all *C. albicans* isolates as equivalent. This is especially important because many mechanistic studies still rely on SC5314. SC5314 is a useful reference strain, but it can show stronger virulence-associated phenotypes than many clinical isolates. For example, SC5314 induced the strongest neutrophil ROS and NET responses among isolates tested in one clinical-isolate panel (Shankar et al., 2020). Clinical *ECE1* variation can also reduce mucosal pathogenicity, and recent vaginal-isolate work shows broad strain-level heterogeneity in *ECE1* expression and pathogenic behavior (Liu et al., 2021; Davari et al., 2026). Interpretation should therefore separate conclusions that are robust across clinical isolates from those driven mainly by SC5314 or another highly virulent background. Second, organotypic systems should be built in modules rather than trying to reproduce the entire ICU environment at once. The minimal clinically useful model should include a polarized epithelial barrier, controlled fungal inoculum, a clinically relevant Candida strain panel, measurable epithelial barrier injury, and antifungal exposure at plausible concentrations. Flow, endothelium, immune cells, or microbiota perturbation should then be added only when they answer a specific route-of-invasion question. Third, prospective cohorts should test candidalysin biomarkers against existing diagnostic and clinical comparators. These comparators should include blood culture, beta-D-glucan, molecular assays, species, source, treatment timing, and outcomes. Cutoffs should be derived in a training or pilot cohort, then locked before independent validation. They should also match the intended use: a high-sensitivity rule-out cutoff, a high-specificity rule-in cutoff, or a prognostic threshold linked to outcomes. Finally, any intervention should enter the clinic only as an adjunct to active antifungal therapy and source control, enriched for toxin-positive *C. albicans* IC.

**Table 1:** Evidence map for candidalysin across the epithelial-systemic interface.

Biological area	Representative finding	How close the evidence is to human IC	Interpretive constraint	Key references
Biogenesis	Hypha-associated *ECE1* expression; precursor processing and conserved secretion sequences	Biochemical/fungal genetics	Expression does not equal secreted active toxin	Moyes et al., 2016; Müller et al., 2024; Lortal et al., 2025
Membrane injury	Polymerization, pore formation, Ca2+ influx, and membrane shedding	Biophysical + cell	Synthetic peptide dose may exceed local *in vivo* exposure	Russell et al., 2022, 2023; Schaefer et al., 2024; Thapa et al., 2026
Host binding	Interactome and sulfated glycosaminoglycan targets	Cell + biochemical	Tissue specificity and receptor redundancy remain	Ikegami et al., 2023; Lin et al., 2024; Zhang et al., 2024
Epithelial barrier	EGFR-MAPK activation, junction failure, and organotypic permeability	Cell/organoid	Mostly mucosal epithelial models; cutaneous evidence is sparse and systemic causality is not established	Ho et al., 2019; Morelli and Queiroz, 2025; Lortal et al., 2026
Gut translocation	Candidalysin-dependent damage permits hypha-associated yeast passage	Organotypic/animal	Critical-illness microbiome and vascular factors incompletely modeled	Allert et al., 2018; Liang et al., 2024; Sprague et al., 2025; Nguyen et al., 2026
Phagocyte escape	NLRP3-GSDMD signaling and NINJ1-dependent rupture cooperate with candidalysin	Cell + animal	Multiple death routes; pathway inhibition may impair host defense	Kasper et al., 2018; Ding et al., 2021; Olivier et al., 2022; Kayagaki et al., 2021; Weerasinghe et al., 2026
Catheter persistence	Candidalysin modulates NETosis and biofilm-host interaction	Device model/animal	Clinical strain and catheter-material diversity	Unger et al., 2023; Tseng et al., 2024; Lee et al., 2025
Human biomarker	Serum anti-candidalysin IgG discriminated pediatric IC in a preliminary cohort	Observational human	Requires adult, prospective, external validation	Luo et al., 2025

The evidence-distance column describes the closest experimental link to human invasive disease; it is not a formal certainty score.

**Table 2:** Candidalysin-directed diagnostic and therapeutic opportunities.

Strategy	Mechanistic rationale	Development status	Key limitation	Recommended next step	Key references
Anti-candidalysin IgG	Serologic footprint of toxin exposure	Partially established diagnostic signal (pediatric cohort)	Background seropositivity and antibody kinetics in adult ICU and colonized patients remain unclear	Define adult ICU baseline distributions and serial changes in a prospective cohort	Luo et al., 2025
Direct peptide assay	Measures active virulence factor	Proposed assay	Signal may be low, transient, bound, or site-restricted	Benchmark stabilized plasma, catheter, and source samples	Sun et al., 2026
Nanobody/antibody	Neutralizes epithelial damage	Preclinical feasibility evidence	Protection is mainly shown in mucosal or non-IC models; systemic exposure and immune tradeoffs are unknown	Test route-specific IC models with echinocandin background, fungal burden, and tissue-injury endpoints	Auffray et al., 2025; Valentine et al., 2024; Yan et al., 2025
GAG mimetic/decoy	Blocks host-surface binding	Proposed therapeutic scaffold from binding studies	Binding specificity must avoid anticoagulation and disruption of endogenous GAG biology	Design non-anticoagulant candidates before efficacy testing	Ikegami et al., 2023; Lin et al., 2024
Production inhibitor	Reduces toxin output	Early discovery anti-virulence lead	Activity may reflect broad hyphal suppression rather than selective candidalysin reduction	Separate *ECE1* output, peptide secretion, morphogenesis, burden, and bypass markers	Kim et al., 2025
Membrane-protective therapy	Enhances repair or peptide shedding	Proposed host-protection concept from cell studies	Host-cell protection could also preserve viable intracellular or adherent fungi	Test only with active antifungal killing and measure burden separately from injury	Russell et al., 2022; Thapa et al., 2026
Inflammasome/NINJ1 modulation	Limits destructive phagocyte rupture	Preclinical host-directed concept	Cytokine and rupture pathways also contribute host defense, leaving a narrow timing window	Define short dosing windows with clearance, cytokine, and tissue-injury rescue endpoints	Lowes et al., 2020; Ding et al., 2021; Kayagaki et al., 2021; Weerasinghe et al., 2026
Detoxified vaccine antigen	Uses candidalysin immunogenicity to prime Th17 defense	Proposed vaccine concept	A detoxified antigen has not yet shown retained immunogenicity without cytolysis	Engineer candidate toxoids and test cytotoxicity loss, antigenicity, and protection	Netea et al., 2015; Nguyen et al., 2026

Early trials should not be powered around mortality before the target is supported in patients. A more realistic first step would prioritize safety, pharmacokinetics, target engagement, and tissue-injury biomarkers. Useful endpoints may include change in free candidalysin, epithelial-injury markers, time to culture clearance, organ-support-free days, and recurrence. Assay development should also address peptide abundance, half-life, compartmentalization, free-versus-bound toxin, and tissue accessibility. Trials should watch for harm: delayed fungal clearance, altered IL-17 responses, or impaired neutrophil function. A platform design could compare neutralization with host-directed modulation while maintaining guideline-concordant background therapy.

## 8 Discussion

Candidalysin remains one of the few fungal virulence factors that can be followed from gene and peptide processing to membrane injury, host signaling, immune remodeling, and disease phenotype. The story has clearly moved beyond a simple pore-forming-toxin model. Candidalysin binds host surface glycans, polymerizes, and provokes membrane repair. It also rewires EGFR signaling and intersects with inflammasome and NINJ1-dependent death pathways. At the host-response level, the toxin reshapes neutrophil responses, travels in biofilm vesicles, and influences both gut commensalism and protective Th17 priming. This breadth is part of what makes the toxin compelling, and it is also why its role is easy to overstate.

Several adjacent areas should now be built into the candidalysin agenda rather than left as background. Comparative Candida pathogenesis is one of them: *C. parapsilosis* can cause important catheter-associated and healthcare-associated IC without relying on candidalysin-like toxin biology, which shows why a toxin-centered model cannot be generalized across species (Kullberg and Arendrup, 2015; Trofa et al., 2008). Host memory and metabolism are another gap. Trained innate responses to Candida have been demonstrated experimentally, while recent macrophage work links nutrient stress to NINJ1-dependent escape; both areas may explain why the same toxin signal produces different outcomes across hosts (Quintin et al., 2012; Weerasinghe et al., 2026). Systems biology approaches, including host-pathogen interactomics, can help connect peptide binding, epithelial signaling, metabolism, and immune remodeling rather than treating each pathway separately (Zhang et al., 2024). Finally, fungal extracellular vesicles should be studied beyond their ability to transport candidalysin, because vesicle cargo and delivery context may change how biofilms communicate with host tissues (Lee et al., 2025).

The field now needs to connect toxin activity, not merely *ECE1* presence, to well-phenotyped invasive disease. Until that link is made, candidalysin-directed strategies are best framed as biomarker-guided adjuncts for selected *C. albicans* infections. That claim is narrower than much of the toxin literature suggests, and better supported by the data now available.
